# Trends and Clinical Impact of Gastrointestinal Endoscopic Procedures on Acute Heart Failure in Spain (2002–2017)

**DOI:** 10.3390/jcm10030546

**Published:** 2021-02-02

**Authors:** Manuel Méndez-Bailón, Rodrigo Jiménez-García, Nuria Muñoz-Rivas, Valentín Hernández-Barrera, José Maria de Miguel-Yanes, Javier de Miguel-Díez, Emmanuel Andrès, Noel Lorenzo-Villalba, Ana López-de-Andrés

**Affiliations:** 1 Internal Medicine Department, Hospital Clínico San Carlos, Universidad Complutense de Madrid, Instituto de Investigación Sanitaria del Hospital Clínico San Carlos (IdISSC), 28040 Madrid, Spain; manuelmenba@hotmail.com; 2 Department of Public Health & Maternal and Child Health, Faculty of Medicine, Universidad Complutense de Madrid, 28040 Madrid, Spain; rodrijim@ucm.es (R.J.-G.); ana.lopez@urjc.es (A.L.-d.-A.); 3 Internal Medicine Department, Hospital Universitario Infanta Leonor, 28031 Madrid, Spain; nmrivas@hotmail.com; 4 Department of Preventive Medicine and Public Health, Faculty of Health Sciences, Universidad Rey Juan Carlos, Alcorcon Madrid, 28922 Madrid, Spain; valentin.hernandez@urjc.es; 5 Internal Medicine Department, Hospital General Gregorio Marañon, 28007 Madrid, Spain; josemaria.demiguel@salud.madrid.org; 6 Respiratory Department, Hospital General Universitario Gregorio Marañón, 28007 Madrid, Spain; jmiguel.hgugm@salud.madrid.org; 7 Service de Médecine Interne, Diabète et Maladies Métaboliques, Hôpitaux Universitaires de Strasbourg, 67000 Strasbourg, France; emmanuel.andres@chru-strasbourg.fr

**Keywords:** heart failure, gastroscopy, colonoscopy

## Abstract

Introduction: Heart failure decompensation can be triggered by many factors, including anemia. In cases of iron deficiency anemia or iron deficiency without anemia, endoscopic studies are recommended to rule out the presence of gastrointestinal neoplasms or other associated bleeding lesions. Objectives: The aims of this study were to (i) examine trends in the incidence, clinical characteristics, and in-hospital outcomes of patients hospitalized with heart failure from 2002 to 2017 who underwent esophagogastroduodenoscopy (EGD) and/or colonoscopy, and to (ii) identify factors associated with in-hospital mortality (IHM) among patients with heart failure who underwent an EGD and/or a colonoscopy. Methods: We conducted an observational retrospective epidemiological study using the Spanish National Hospital Discharge Database (SNHDD) between 2002 and 2017. We included hospitalizations of patients with a primary discharge diagnosis of heart failure. Cases were reviewed if there was an ICD-9-CM or ICD-10 procedure code for EGD or colonoscopy in any procedure field. Multivariable logistic regression models were constructed to identify predictors of IHM among HF patients who underwent an EGD or colonoscopy. Results: A total of 51,187 (1.32%) non-surgical patients hospitalized with heart failure underwent an EGD and another 72,076 (1.85%) patients had a colonoscopy during their admission. IHM was significantly higher in those who underwent an EGD than in those who underwent a red blood cell transfusion (OR 1.10; 95%CI 1.04–1.12). However, the use of colonoscopy seems to decrease the probability of IHM (OR 0.45; 95%CI 0.41–0.49). In patients who underwent a colonoscopy, older age seems to increase the probability of IHM. However, EGD was associated with a lower mortality (OR 0.60; 95% CI 0.55–0.64). Conclusion: In our study, a decrease in the number of gastroscopies was observed in relation to colonoscopy in patients with heart failure. The significant ageing of the hospitalized HF population seen over the course of the study could have contributed to this. Both procedures seemed to be associated with lower in-hospital mortality, but in the case of colonoscopy, the risk of in-hospital mortality was higher in elderly patients with heart failure and associated neoplasms. Colonoscopy and EGD seemed not to increase IHM in patients with heart failure.

## 1. Introduction

Acute heart failure (HF) is a common and potentially fatal condition and is one of the most frequent causes of hospitalization worldwide. The prognosis is still poor, despite improvements in diagnosis and overall management [1,2]. Heart failure decompensation can be triggered by anemia or weight loss, along with deterioration of the patient’s overall general condition. These clinical situations are usually encountered in elderly patients with heart failure with preserved ejection fraction. Many patients with heart failure use antithrombotic treatments, including antiplatelet and/or anticoagulant agents.

The prevalence of anemia in patients with HF is variable and ranges from 9% to 69.6%, depending on the definition used and the population studied [3,4]. The prevalence has been reported to reach approximately 30% in stable and 50% in hospitalized patients, regardless of whether patients have heart failure with reduced ejection fraction (HFrE) or HF with preserved ejection fraction, compared with < 10% in the general population. The prevalence of HF increases with age, exceeding 20% in patients ≥85 years old [5].

The pathogenesis of anemia in HF is multifactorial, with iron deficiency (ID) more frequently encountered than vitamin B12 and folic acid deficiency. Iron deficiency is a very common comorbidity in patients with heart failure, regardless of race, sex, anemia, and left ventricular ejection fraction (LVEF) [6,7]. Low levels of available iron have been reported in nearly 50% of patients with HF, with or without anemia, who have low levels of available iron [8,9].

Endoscopic studies are recommended in patients with iron deficiency anemia to rule out the presence of gastrointestinal (GI) neoplasms or any other type of associated bleeding lesions. Many patients with HF are elderly and have associated risk factors such as obesity or diabetes that increase the incidence of neoplasms, including GI tumors. GI changes associated with heart failure have been investigated in several studies [10,11], and include mosaic patterns in the stomach, mucosal thickening, antral vascular ectasia, and areas of telangiectasias, among others [11].

Clinicians are faced with the decision of whether to perform endoscopic studies in patients with HF. However, there is little evidence to assess the clinical impact of performing endoscopic studies in patients hospitalized for heart failure in our setting.

The objectives of this study were to (i) examine trends in the incidence, clinical characteristics, and in-hospital outcomes of patients hospitalized with heart failure from 2002 to 2017 who underwent EGD and/or colonoscopy, and to (ii) identify factors associated with in-hospital mortality (IHM) among patients with heart failure who underwent EGD or colonoscopy.

## 2. Materials and Methods

### 2.1. Design, Setting and Participants

We designed a retrospective observational investigation obtaining data from the Spanish National Hospital Discharge Database (SNHDD) [12]. The study time-period included all of the years from 2002 to 2017. The SNHDD collects information for each hospital admission, including patient’s characteristics (sex and age), diagnosis, and procedures conducted during the hospital stay, as well as in-hospital mortality (IHM). The International Classification of Diseases Ninth Revision (ICD-9CM) was used by the SNHDD for coding from 2002 to 2015 and the Tenth Revision (ICD-10) for years 2016 and 2017.

The study population included adults (≥ 18 years) that, according to the criteria of the American College of Cardiology/American Heart Association [13], suffered heart failure, using the codes listed in Table S1. These codes had to be recorded as the primary diagnosis.

Patients who had undergone surgery were excluded using the algorisms described by Metha et al. [14].

The codes for EGD and colonoscopy (Table S1) recorded in any procedure position allowed us to create two cohorts of patients, one for each procedure.

### 2.2. Study Variables

The study variables included patients’ sex and age, the presence of concomitant chronic conditions, length of hospital stay (LOHS), and IHM. The comorbid conditions analyzed were those included in the Charlson Comorbidity Index (CCI), identified using the algorithms proposed by Quan et al. [15]. The CCI was analyzed as a continuous variable. We also showed the specific prevalence of the following conditions: ischemic coronary disease, atrial fibrillation, anemia, type 2 diabetes mellitus (T2DM), chronic liver disease, angiodysplasia, acute renal failure, chronic obstructive pulmonary disease (COPD), colon cancer, stomach cancer, GI bleeding, and inflammatory bowel disease in any diagnosis field, as well as red blood cell transfusion in any procedure field. The ICD codes are shown in Table S1.

### 2.3. PSM Method

We obtained propensity scores (PS) for HF patients who underwent an EGD study and those who did not, as well as for those who underwent a colonoscopy and those who did not [16]. The PS matching (PSM) method consisted of selecting HF patients who underwent an EGD and/or colonoscopy, and a population of HF patients without any endoscopic study in their discharge report with the same or a similar PS [16,17]. The variables included in each PSM model were year of admission, sex, age, CCI, and all of the analyzed comorbidities. In order to assess the isolated effect of EGD and colonoscopy, we also matched for this variable. The PS was calculated using multivariate logistic regression.

### 2.4. Statistical Methods

For the study purposes, the period from 2002 to 2017 was divided into eight two-year periods. Incidence of hospitalizations with heart failure was calculated using the population by age groups and sex provided by the Instituto National de Estadistica (Spanish National Institute of Statistics) for each two years period as the denominator [12]. The Poisson regression method was applied to assess significant changes overtime after adjusting for changes in the distribution of the population according to age and sex.

Descriptive statistics for categorical variables included counts and proportions, as well as means with standard deviations, for continuous variables.

To assess possible changes in categorical and continuous variables from 2002 to 2017, the χ2 t for linear trend and ANOVA were the statistical tests used.

McNemar’s test and the paired t-test were used to compare the study groups (EGD vs. no EGD and colonoscopy vs. no colonoscopy) after PSM [18]. Variables independently associated with IHM in patients hospitalized with heart failure who received an EGD or a colonoscopy were identified by constructing multivariate logistic regression models. Stata version 14 (Stata, College Station, Texas, USA) was used for the data analysis.

### 2.5. Sensitivity Analysis

In order to control the confounding effect of gastrointestinal cancer on the performance of an endoscopic procedure, we conducted a sensitivity analysis. To do so, we excluded those patients who had a code for stomach or colon cancer from the study, and re-analyzed the database using PSM as described in Section 2.3.

### 2.6. Ethical Aspects

According to Spanish legislation, ethical approval by an ethics committee is not required when investigations are conducted with public access anonymized administrative databases.

## 3. Results

### 3.1. Heart Failure Hospitalizations

As can be seen in Table 1, the number of hospitalizations in Spain with an HF diagnosis was over 3.8 million between 2002 and 2017. The incidence of heart failure coding and age increased significantly over time, and the percentage of women decreased over the study period (53.15% vs. 52.2%; *p* < 0.001; Table 1).

**Figure 1:**
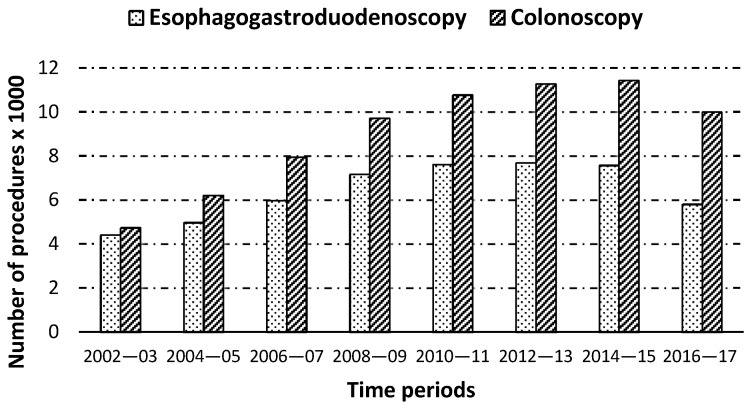
Number of eophagogastroduodenoscopies and colonoscopies conducted on patients hospitalized suffering heart failure in Spain from 2002 to 2017.

The proportion of patients with HF who underwent an EGD significantly decreased from the period of 2002–2003 to the period of 2016–2017 (1.37% vs. 0.94%; *p* < 0.001). However, the time trend for colonoscopy shows a small, but significant, (*p* < 0.01) increase from 2002–2003 (1.47%) to 2016–2017 (1.62%). The total number of procedures conducted for each time period is shown in Figure 1.

We found a significant increase in comorbidity according to the mean CCI over time (2.19 ± 0.98 in 2002–2003 vs. 2.49 ± 1.11 in 2016–2017; *p* < 0.001). The most common associated comorbidities for hospitalized patients for heart failure were atrial fibrillation (43.4%), T2DM (35.11%), and acute renal failure (25.07%). There was a significant decrease in the frequency of the use of red blood cell transfusions (from 6.2% in 2002–2003 to 5.42% in 2016–2017; *p* < 0.001).

The mean LOHS for admissions of heart failure was 11.29 days in the period of 2002–2003, decreasing to 9.78 days in 2016–2017 (*p* < 0.001). For the total time period, the crude IHM was 12.73%. The crude IHM decreased significantly over time from 13.79% in 2002–2003 to 12.41% in 2016–2017 (*p* < 0.001; Table 1).

### 3.2. Distribution of Study Covariates among Patients Hospitalized with Heart Failure with or without a Gastroscopy

According to the SNHDD, 51,187 (1.32%) non-surgical patients hospitalized with heart failure had undergone an EGD in Spain from 2002 to 2017. Table 2 shows the distribution of the variables studied according to the performance or nonperformance of EGD before and after PSM.

Before matching, the prevalence of colonoscopy in our population (32.28% vs. 1.45% *p* < 0.001) was higher among patients who underwent EGD. However, when PSM was conducted with the inclusion of colonoscopy as a matching variable, the difference between both groups was not significant. When we compared patients that underwent an EGD with matched controls after PSM, we found significantly lower rates of red blood cell transfusion in patients who underwent EGD (41.47% and 43.19%, respectively; *p* < 0.001).

After PSM, IHM during admission for heart failure was 9.68% in participants who underwent EGD and 14.97% in the matched controls (*p* < 0.001) who did not undergo this procedure. The mean LOHS was 17.51 ± 16.08 days among people who underwent EGD and 14.87 ± 14.36 days among matched controls (*p* < 0.001).

### 3.3. Distribution of Study Covariates among Patients Hospitalized with Heart Failure That Did or Did Not Undergo Colonoscopy

In Spain from 2002 to 2017, a total of 72,076 (1.85%) patients hospitalized for heart failure underwent colonoscopies during their admission. Table 3 shows the distribution of the study variables among patients, related to colonoscopy before and after PSM.

In our study sample, when PSM was conducted, the prevalence of EGD (22.92% vs. 20.81% *p* < 0.001) was slightly higher among patients who underwent a colonoscopy. However, we found significantly lower rates of red cell transfusions in patients receiving a colonoscopy (36.58% and 38.62%, respectively; *p* < 0.001) compared to the matched controls.

After PSM, IHM during admission for heart failure was 7.87% in patients who underwent a colonoscopy and 16.22% in the matched controls (*p* < 0.001). The mean LOHS was 18.56 ± 16.43 days among those who underwent a colonoscopy and 13.68 ± 12.1 days among the matched controls (*p* < 0.001).

### 3.4. Multivariable Logistic Regression Analysis of the Factors Associated with IHM in Patients Hospitalized with Heart Failure Who Underwent Gastroscopy or Colonoscopy

As shown in Table 4, for patients who underwent an EGD or colonoscopy, IHM decreased significantly over time. IHM was significantly higher in those with comorbidities such as colon cancer, stomach cancer, GI bleeding, and inflammatory bowel disease. Female sex decreased the probability of dying in both procedures.

Among those who underwent EGD, IHM was significantly higher in those who received a red blood cell transfusion (OR 1.1; 95%CI 1.04–1.117). However, the use of colonoscopy decreased the probability of dying (OR 0.45; 95% 0.41–0.49). In patients who underwent a colonoscopy, older age increased the probability of dying. However, undergoing a gastroscopy was associated with a lower mortality (OR 0.6; 95%CI 0.55–0.64).

### 3.5. Sensitivity Analysis

The results of the PSM after excluding patients who had a code for stomach or colon cancer are shown in Tables S2 and S3. The IHM among patients suffering HF who underwent EGD was significantly lower than among the matched HF patients who did not receive this procedure (8.71% vs. 13.91%; *p* < 0.001). After PSM, the same difference was observed for colonoscopy (7.60% vs. 15.46% *p* < 0.001).

## 4. Discussion

This study showed a decrease in endoscopic procedures being performed throughout the follow-up period in patients with heart failure. These findings may be due to a decrease in the incidence of upper GI bleeding as a cause of anemia in recent years, greater use of gastric protectors such as omeprazole, and a decrease in the use of non-steroidal anti-inflammatory drugs or anti-aggregants at high doses [19,20,21].

There has not been a slight and steady increase in the use of colonoscopy in HF patients. This could be the result of physicians being more or less proactive in conducting endoscopic studies to rule out the presence of GI neoplasia, primarily colon cancer, in this elderly population. The presence of a failing heart, per se, might contribute to tumor progression and formation. Epidemiological data have emerged that HF patients are at higher risk to be diagnosed with, and/or to die from, cancer compared with age-matched subjects without HF [22]. Cardiovascular disease (CVD; including HF) and cancer share pathophysiologic similarities and possible interactions, including several common risk factors, suggesting similar trigger mechanisms [23]. Diabetes, hypertension, obesity, smoking, diet, inflammation, oxidative stress, and physical inactivity are all contributors to the development of both CVD (HF) and cancer [24].

In our study, the incidence of colon cancer was higher than gastric cancer. These findings may justify the increased use of colonoscopy during heart failure patient hospitalization. Furthermore, the presence of iron deficiency anemia could affect up to 50% of hospitalized patients with HF. The European Society of Cardiology (ESC) guidelines for heart failure recommend that patients with iron deficiency should be screened for any potentially reversible or treatable causes (Class IC level of evidence) [25]. The clinical practice guidelines for the management of iron deficiency anemia recommend performing a colonoscopy study in patients aged ≥ 50 years, which may justify the greater use of colonoscopy observed in our work. In the literature, only 40–50% of patients with iron-deficient anemia undergo an endoscopic workup [26,27]. Martens et al. also showed that patients with iron deficiency but without anemia still had a significant risk of having an underlying GI malignancy [28].

In this study, the most common associated conditions to heart failure were atrial fibrillation and chronic renal disease. Both entities are related to the development of anemia in patients with HF. Atrial fibrillation requires the use of oral anticoagulation for its management, which can lead to an increased risk of GI bleeding. Erythropoietin levels are increased in proportion to HF severity but are lower than expected for the degree of anemia, suggesting blunted erythropoietin production [29,30].

Patients with heart failure and kidney failure frequently develop occult GI bleeding because of the presence of angiodysplasias, which increased during the study period.

The differences between EGD and colonoscopy could likely be related to the different indications for these procedures (e.g., bleeding type if overt, upper gastrointestinal vs. lower gastrointestinal symptoms) rather than to the crude incidence of gastric and colon cancer. The significant ageing of the hospitalized HF population seen over the course of the study could also have contributed to it.

Another significant finding was the decrease in the use of red blood cell transfusions in our population. This finding may be due to a more restrictive indication for the use of transfusions in this type of patient, as recommended by clinical practice guidelines [25,31], and to the use of intravenous iron as an alternative to transfusion. In numerous clinical trials, the use of intravenous iron has demonstrated efficacy and safety for increasing hemoglobin values in patients with HF and reduced ejection fraction. The AFFIRM-AHF clinical trial has also shown the benefit for the use of intravenous iron in a hospital setting in patients with decompensated HF [32].

In relation to the use of blood transfusions in patients with GI bleeding, the published clinical practice guidelines are more restrictive. These recommendations may have been reflected in our findings, with lower transfusion rates in patients undergoing gastroscopy.

Gastroscopy seems to be safe and associated with lower in-hospital mortality. These findings may be because this procedure allows for control of upper GI bleeding in patients with HF on anticoagulant and antiplatelet therapy. The higher mortality observed in patients in which GI endoscopy is not performed may be attributable to the older age of the patients, as can be observed in the adjustment for propensity matching score. In this group of patients in which the procedure was indicated, it was likely not performed because of the risk associated with procedural sedation.

Colonoscopies were a relatively safe procedure to perform in patients with HF. This procedure was associated with a lower in-hospital mortality rate in patients admitted for heart failure. In contrast, Abu et al. concluded that severe heart failure patients were at high risk for mortality and complications after GI procedures. Their study included 207 patients with severe heart failure, with a mean age of 64.8 years (younger than ours). In their cohort, 45.5% presented with heart failure exacerbated by fluid overload. Mortality within 30 days after the procedure was 4.3%, and the 30-day complication incidence was 19.8% [33].

Patients who underwent an EGD or colonoscopy seemed to have a lower mortality than those who did not, probably for one of the following reasons: (1) The rapid development of technological advances in gastrointestinal endoscopy has enhanced the gastroenterologist’s ability to diagnose/treat lesions and avoid/reduce complications derived from these procedures within the gastrointestinal tract. The basic endoscope has remained similar in its fundamental design since its early adoption, but technologies from other scientific disciplines and collaborative work within the engineering community allow for it to safely and effectively accomplish its evolving remit. (2) The availability of anesthesia. (3) Patients undergoing these procedures are in better clinical condition, and the procedures are not retained in those with advanced or unsteady disease. (4) In some countries, the development of multidisciplinary units for the management of patients with heart failure [34,35,36,37,38,39].

This study has some implications on gastroenterology practice, namely: (1) there is a profile of patients with heart failure where the risk of peri-procedure mortality may be increased, such as older subjects with associated comorbidities and GI neoplasms. In this group of patients, the risk of in-hospital mortality is increased, but may also be attributable to a higher risk of complications, such as intestinal perforation and subsequently GI bleeding. The indication for endoscopic studies in elderly frail HF patients with associated comorbidities should be individualized. (2) In patients with high peri-procedure risk, other less invasive diagnostic tools, such as capsule endoscopy, virtual colonoscopy, and immunohistochemical fecal occult blood tests, could be considered [40].

The present study has some limitations. (1) It was a retrospective study, so we could not establish a cause-effect relationship between gastroscopy/colonoscopy and the survival of patients with HF. (2) The lack of relevant clinical variables, such as left ventricular ejection fraction, pro-BNP levels, hemoglobin levels, iron, or other therapies administered, made interpretation difficult. (3) The types of heart failure (systolic or diastolic) were unknown, and this is important as the risk of adverse events and death may be higher among those who have systolic CHF compared with diastolic heart failure. (4) There were significant confounding factors that could influence the IHM (e.g., patients with malignancy were also included). However, the figures of IHM observed after excluding patients with stomach and colon cancer, as part of a sensitivity analysis, were very similar to those found for the entire study population, suggesting that the effect of these conditions on our results, if any, was of a small magnitude. (5) Post endoscopy complications were not available. (6) Changes in the ICD used for coding (ICD9 from 2002–2015 and ICD10 in 2016–2017) could have resulted in significant variation over time in some conditions, such as chronic liver disease, COPD, and red cell transfusion, for which we observed a jump in the prevalence from 2002–2015 to 2016–2017. However, as can be seen in Table 1, beside these three conditions, for all of the rest of conditions analyzed, the time trend showed a logical evolution over time, proving, in our opinion, that our investigation, even with the change from ICD 9 to ICD 10, provides reliable data. In any case, as for the main objective of this investigation, we used a PSM method for the changes in coding, which equally affected those who underwent a gastroscopy or a colonoscopy, as well as those who did not, so the information bias would be non-differential and would therefore not affect the main results of our study. (7) The methodology used could only show associations, and the reason for these associations would require further testing. Therefore, this is currently one of the largest epidemiological studies regarding GI endoscopies in HF. According to our results, these procedures seem to be safe in patients with HF, but special attention should be given to elderly individuals with comorbid conditions. However, we cannot make solid conclusions based on this study.

According to our results, prospective studies aimed at evaluating the impact of gastrointestinal procedures (diagnostic rentability and procedure-related complications) on patients with heart failure will be necessary.

## 5. Conclusions

In our study, a decrease in the number of gastroscopies is observed in relation to colonoscopies in patients with heart failure. The significant ageing of the hospitalized HF population seen over the course of the study could also have contributed to it. Both procedures seem to be associated with a lower in-hospital mortality, but in the case of colonoscopy, the risk of in-hospital mortality was higher in elderly patients with heart failure and associated neoplasms. Colonoscopy and EGD seem to not increase IHM in patients with heart failure.

## Figures and Tables

**Table 1:** Characteristics of hospital admissions with a diagnosis of heart failure in Spain (2002–2017), including the use of endoscopic studies.

Variables	2002–2003	2004–2005	2006–2007	2008–2009	2010–2011	2012–2013	2014–2015	2016–2017	Total
Number of hospital admissions	322,404	374,205	419,752	472,706	516,195	559,658	608,784	617,914	3891,618
Incidence per 100,000 population *	468.96	524.85	568.31	621.88	673.07	730.20	799.05	809.85	653.24
Female sex, *n* (%) *	171,366 (53.15)	197,542 (52.79)	221,102 (52.67)	249,179 (52.71)	270,879 (52.48)	292,693 (52.3)	316,917 (52.06)	322,561 (52.2)	2042,239 (52.48)
Age, years, mean (SD) *	76.49 (10.9)	76.92 (10.86)	77.46 (10.9)	78.01 (10.84)	78.61 (10.77)	79.09 (10.75)	79.54 (10.77)	80.11 (10.77)	78.53 (10.87)
< 60 years, *n* (%) *	23,924 (7.42)	26,848 (7.17)	29,295 (6.98)	30,665 (6.49)	31,264 (6.06)	32,826 (5.87)	34,348 (5.64)	32,953 (5.33)	242,123 (6.22)
60–75 years, *n* (%) *	105,639 (32.77)	115,387 (30.84)	119,520 (28.47)	124,686 (26.38)	124,547 (24.13)	125,540 (22.43)	133,222 (21.88)	130,904 (21.18)	979,445 (25.17)
76–85 years, *n* (%) *	129,197 (40.07)	155,867 (41.65)	178,213 (42.46)	202,388 (42.81)	221,751 (42.96)	238,998 (42.7)	249,420 (40.97)	241,085 (39.02)	1616,919 (41.55)
> 85 years, *n* (%) *	63,644 (19.74)	76,103 (20.34)	92,724 (22.09)	114,967 (24.32)	138,633 (26.86)	162,294 (29)	191,794 (31.5)	212,972 (34.47)	1053,131 (27.06)
EGD, *n* (%) *	4409 (1.37)	4972 (1.33)	5969 (1.42)	7166 (1.52)	7612 (1.47)	7687 (1.37)	7568 (1.24)	5804 (0.94)	51,187 (1.32)
Colonoscopy, *n* (%) *	4739 (1.47)	6213 (1.66)	7953 (1.89)	9712 (2.05)	10,768 (2.09)	11,267 (2.01)	11,428 (1.88)	9996 (1.62)	72,076 (1.85)
CCI, mean (SD) *	2.19 (0.98)	2.28 (1.01)	2.3 (1.01)	2.35 (1.02)	2.39 (1.02)	2.44 (1.04)	2.46 (1.04)	2.49 (1.11)	2.38 (1.04)
Ischemic coronary disease, *n* (%) *	97,548 (30.26)	114,861 (30.69)	124,593 (29.68)	136,082 (28.79)	141,906 (27.49)	152,626 (27.27)	159,558 (26.21)	153,259 (24.8)	1080,433 (27.76)
Atrial fibrillation, *n* (%) *	119,835 (37.17)	147,922 (39.53)	172,769 (41.16)	200,508 (42.42)	224,191 (43.43)	250,202 (44.71)	277,822 (45.64)	295,617 (47.84)	1688,866 (43.4)
Anemia, *n* (%) *	719 (0.22)	871 (0.23)	1068 (0.25)	1316 (0.28)	1497 (0.29)	1741 (0.31)	1828 (0.30)	1789 (0.29)	10,829 (0.28)
Chronic liver disease, *n* (%) *	8949 (2.78)	11,538 (3.08)	13,449 (3.2)	15,531 (3.29)	17,488 (3.39)	20,229 (3.61)	22,984 (3.78)	12,201 (1.97)	122,369 (3.14)
Angiodysplasia, *n* (%)	965 (0.3)	1478 (0.39)	1894 (0.45)	2402 (0.51)	3054 (0.59)	3618 (0.65)	4128 (0.68)	4445 (0.72)	21,984 (0.56)
Acute renal failure, *n* (%) *	49,726 (15.42)	66,197 (17.69)	80,701 (19.23)	104,535 (22.11)	128,249 (24.85)	156,387 (27.94)	184,424 (30.29)	205,242 (33.22)	975,461 (25.07)
T2DM, *n* (%) *	95,182 (29.52)	122,448 (32.72)	142,169 (33.87)	166,936 (35.31)	185,036 (35.85)	202,878 (36.25)	220,319 (36.19)	231,517 (37.47)	1366,485 (35.11)
COPD, *n* (%) *	71,136 (22.06)	81,937 (21.9)	82,076 (19.55)	89,205 (18.87)	97,518 (18.89)	104,762 (18.72)	111,752 (18.36)	171,698 (27.79)	810,084 (20.82)
Colon cancer, *n* (%) *	1413 (0.44)	1960 (0.52)	2328 (0.55)	2817 (0.6)	3187 (0.62)	3674 (0.66)	3999 (0.66)	4152 (0.67)	23,530 (0.6)
Stomach cancer, *n* (%) *	702 (0.22)	789 (0.21)	905 (0.22)	1067 (0.23)	1164 (0.23)	1191 (0.21)	1237 (0.2)	1235 (0.2)	8290 (0.21)
Gastrointestinal bleeding, *n* (%) *	6074 (1.88)	7167 (1.92)	7963 (1.9)	8888 (1.88)	9713 (1.88)	10,333 (1.85)	11,134 (1.83)	13,201 (2.14)	74,473 (1.91)
Inflammatory bowel disease, *n* (%) *	556 (0.17)	708 (0.19)	861 (0.21)	1100 (0.23)	1212 (0.23)	1430 (0.26)	1828 (0.3)	1958 (0.32)	9653 (0.25)
Red cell transfusion, *n* (%) *	19,991 (6.2)	24,620 (6.58)	30,054 (7.16)	36,601 (7.74)	42,832 (8.3)	47,414 (8.47)	50,074 (8.23)	33,505 (5.42)	285,091 (7.33)
IHM, *n* (%) *	44,461 (13.79)	49,725 (13.29)	54,075 (12.88)	60,551 (12.81)	65,041 (12.6)	69,594 (12.44)	75,129 (12.34)	76,670 (12.41)	495.246 (12.73)
LOHS, mean (SD)	11.29 (10.94)	11.12 (10.85)	10.89 (10.47)	10.74 (10.57)	10.32 (10.24)	9.84 (9.29)	9.74 (9.25)	9.78 (9.38)	10.34 (10.35)

EGD—esophagogastroduodenoscopy; CCI—Charlson Comorbidity Index; T2DM—type 2 diabetes mellitus; COPD—chronic obstructive pulmonary disease; LOHS—length of hospital stay; IHM—in hospital mortality; * *p* < 0.001 to assess time trend from 2002 to 2017.

**Table 2:** Distribution of the variables studied according to the performance or nonperformance of esophagogastroduodenoscopy: crude and propensity score-matched data (2002–2017).

Variables	Before Matching			After Matching		
	EGD	No EGD	*p*-Value	EGD	No EGD	*p*-Value
2002–2003, *n* (%)	4409 (8.61)	317,995 (8.28)	<0.001	4409 (8.61)	4632 (9.05)	0.083
2004–2005, *n* (%)	4972 (9.71)	369,233 (9.61)		4972 (9.71)	5156 (10.07)	
2006–2007, *n* (%)	5969 (11.66)	413,783 (10.77)		5969 (11.66)	6024 (11.77)	
2008–2009, *n* (%)	7166 (14)	465,540 (12.12)		7166 (14)	7010 (13.69)	
2010–2011, *n* (%)	7612 (14.87)	508,583 (13.24)		7612 (14.87)	7532 (14.71)	
2012–2013, *n* (%)	7687 (15.02)	551,971 (14.37)		7687 (15.02)	7572 (14.79)	
2014–2015, *n* (%)	7568 (14.79)	601,216 (15.65)		7568 (14.79)	7507 (14.67)	
2016–2017, *n* (%)	5804 (11.34)	612,110 (15.94)		5804 (11.34)	5754 (11.24)	
Female sex, *n* (%)	25715 (50.24)	2016,524 (52.51)	<0.001	25,715 (50.24)	25,949 (50.69)	0.144
Age, years, and mean (SD)	77.51 (9.68)	78.54 (10.88)	<0.001	77.51 (9.68)	77.71 (9.94)	<0.001
< 60 years, *n* (%)	2753 (5.38)	239,370 (6.23)	<0.001	2753 (5.38)	2782 (5.43)	<0.001
60–75 years, *n* (%)	14,473 (28.27)	964,972 (25.13)		14,473 (28.27)	14,060 (27.47)	
76–85 years, *n* (%)	24,642 (48.14)	1592,277 (41.46)		24,642 (48.14)	24,344 (47.56)	
85 years, *n* (%)	9319 (18.21)	1043,812 (27.18)		9319 (18.21)	10,001 (19.54)	
Colonoscopy, *n* (%)	16,521 (32.28)	55,555 (1.45)	<0.001	16,521 (32.28)	16,230 (31.71)	0.051
CCI, mean (SD)	2.5 (1.06)	2.38 (1.04)	<0.001	2.5 (1.06)	2.41 (1.03)	<0.001
Ischemic coronary disease, *n* (%)	11,595 (22.65)	1068,838 (27.83)	<0.001	11,595 (22.65)	10,746 (20.99)	<0.001
Atrial fibrillation, *n* (%)	21,575 (42.15)	1667,291 (43.41)	<0.001	21,575 (42.15)	21,691 (42.38)	0.463
Anemia, *n* (%)	312 (0.61)	10,517 (0.27)	0.001	310 (0.61)	331 (0.66)	0.405
Chronic liver disease, *n* (%)	4551 (8.89)	117,818 (3.07)	<0.001	4551 (8.89)	4786 (9.35)	<0.001
Angiodysplasia, *n* (%)	4023 (7.86)	17,961 (0.47)	<0.001	4023 (7.86)	3236 (6.32)	0.355
Acute renal failure, *n* (%)	13,096 (25.58)	962,365 (25.06)	0.006	13,096 (25.58)	12,967 (25.33)	0.002
Type 2 diabetes, *n* (%)	17,023 (33.26)	1349,462 (35.14)	<0.001	17,023 (33.26)	16,564 (32.36)	<0.001
COPD, *n* (%)	8990 (17.56)	801,094 (20.86)	<0.001	8990 (17.56)	8399 (16.41)	0.051
Colon cancer, *n* (%)	817 (1.6)	22,713 (0.59)	<0.001	817 (1.6)	897 (1.75)	0.743
Stomach cancer, *n* (%)	1385 (2.71)	6905 (0.18)	<0.001	1385 (2.71)	1368 (2.67)	0.038
Gastrointestinal bleeding, *n* (%)	11,554 (22.57)	62,919 (1.64)	<0.001	11,554 (22.57)	11,832 (23.12)	0.517
Inflammatory bowel disease, *n* (%)	166 (0.32)	9487 (0.25)	<0.001	166 (0.32)	178 (0.35)	<0.001
Red cell transfusion, *n* (%)	21,229 (41.47)	263,862 (6.87)	<0.001	21,229 (41.47)	22,110 (43.19)	<0.001
LOHS, *n* (%)	17.51 (16.08)	10.25 (10.24)	<0.001	17.51 (16.08)	14.87 (14.36)	<0.001
IHM, *n* (%)	4956 (9.68)	490,290 (12.77)	<0.001	4956 (9.68)	7662 (14.97)	<0.001

CCI—Charlson Comorbidity Index; COPD—chronic obstructive pulmonary disease, LOHS—length of hospital stay; IHM—in hospital mortality.

**Table 3:** Characteristics of patients hospitalized with heart failure according to the performance or nonperformance of colonoscopy: crude and propensity score adjusted data (2002–2017).

Variables	Before Matching			After Matching		
	Colonoscopy	No Colonoscopy	*p*-Value	Colonoscopy	No Colonoscopy	*p*-Value
2002–2003, *n* (%)	4739 (6.58)	317,665 (8.32)	<0.001	4739 (6.58)	5126 (7.11)	<0.001
2004–2005, *n* (%)	6213 (8.62)	367,992 (9.63)		6213 (8.62)	6482 (8.99)	
2006–2007, *n* (%)	7953 (11.03)	411,799 (10.78)		7953 (11.03)	7996 (11.09)	
2008–2009, *n* (%)	9712 (13.47)	462,994 (12.12)		9712 (13.47)	9482 (13.16)	
2010–2011, *n* (%)	10,768 (14.94)	505,427 (13.23)		10,768 (14.94)	10,395 (14.42)	
2012–2013, *n* (%)	11,267 (15.63)	548,391 (14.36)		11,267 (15.63)	10,942 (15.18)	
2014–2015, *n* (%)	11,428 (15.86)	597,356 (15.64)		11,428 (15.86)	11,205 (15.55)	
2016–2017, *n* (%)	9996 (13.87)	607,918 (15.92)		9996 (13.87)	10,448 (14.5)	
Female sex, *n* (%)	36,626 (50.82)	2005,613 (52.51)	<0.001	36,626 (50.82)	36,564 (50.73)	0.744
Age, years, mean (SD)	77.65 (9.14)	78.54 (10.9)	<0.001	77.65 (9.14)	77.72 (9.79)	0.818
< 60 years, *n* (%)	3254 (4.51)	238,869 (6.25)	<0.001	3254 (4.51)	3686 (5.11)	<0.001
60–75 years, *n* (%)	20,558 (28.52)	958,887 (25.1)		20,558 (28.52)	19,904 (27.62)	
76–85 years, *n* (%)	35,765 (49.62)	1581,154 (41.4)		35,765 (49.62)	34,658 (48.09)	
> 85 years, *n* (%)	12,499 (17.34)	1040,632 (27.24)		12,499 (17.34)	13,828 (19.19)	
EGD, *n* (%)	16,521 (22.92)	34,666 (0.91)	<0.001	16,521 (22.92)	14,996 (20.81)	<0.001
CCI, mean (SD)	2.30 (1.03)	2.38 (1.04)	<0.001	2.30 (1.03)	2.41 (1.05)	<0.001
Ischemic coronary disease, *n* (%)	16,587 (23.01)	1063,846 (27.85)	<0.001	16,587 (23.01)	15,500 (21.51)	<0.001
Atrial fibrillation, *n* (%)	30,979 (42.98)	1657,887 (43.41)	0.023	30,979 (42.98)	30,602 (42.46)	0.045
Anemia, *n* (%)	480 (0.71)	10,317 (0.27)	<0.001	509 (0.71)	525 (0.73)	<0.662
Chronic liver disease, *n* (%)	4349 (6.03)	118,020 (3.09)	<0.001	4349 (6.03)	4690 (6.51)	<0.001
Angiodysplasia, *n* (%)	4300 (5.97)	17,684 (0.46)	<0.001	4300 (5.97)	3856 (5.35)	<0.001
Acute renal failure, *n* (%)	19,022 (26.39)	956,439 (25.04)	<0.001	19,022 (26.39)	18,764 (26.03)	0.122
Type 2 diabetes, *n* (%)	25,478 (35.35)	1341,007 (35.11)	0.182	25,478 (35.35)	24,592 (34.12)	0.182
COPD, *n* (%)	13,383 (18.57)	796,701 (20.86)	<0.001	13,383 (18.57)	12,750 (17.69)	<0.001
Colon cancer, *n* (%)	4711 (6.54)	18,819 (0.49)	<0.001	4711 (6.54)	5099 (7.07)	<0.001
Stomach cancer, *n* (%)	390 (0.54)	7900 (0.21)	<0.001	390 (0.54)	513 (0.71)	<0.001
Gastrointestinal bleeding, *n* (%)	14,997 (20.81)	59,476 (1.56)	<0.001	14,997 (20.81)	15,495 (21.5)	<0.001
Inflammatory bowel disease, *n* (%)	996 (1.38)	8657 (0.23)	<0.001	996 (1.38)	1179 (1.64)	<0.001
Red cell transfusion, *n* (%)	26,369 (36.58)	258,722 (6.77)	<0.001	26,369 (36.58)	27,837 (38.62)	<0.001
LOHS, *n* (%)	18.56 (16.43)	10.19 (10.18)	<0.001	18.56 (16.43)	13.68 (12.1)	<0.001
IHM, *n* (%)	5672 (7.87)	48,9574 (12.82)	<0.001	5672 (7.87)	11,688 (16.22)	<0.001

EGD—esophagogastroduodenoscopy; CCI—Charlson Comorbidity Index; COPD—chronic obstructive pulmonary disease; LOHS—length of hospital stay; IHM—in hospital mortality.

**Table 4:** Logistic regression analysis of factors associated with in-hospital mortality in patients with heart failure who underwent an EGD and/or colonoscopy in Spain, 2002–2017.

Variables	EGD OR (95%CI)	Colonoscopy OR (95%CI)
Year	0.97 (0.96–0.99)	0.93 (0.92–0.94)
Female sex	0.83 (0.77–0.88)	0.82 (0.77–0.87)
60–75 years,	0.77 (0.67–0.87)	1.03 (0.89–1.19)
76–85 years	0.86 (0.76–0.98)	1.12 (0.97–1.28)
>85 years	1.03 (0.9–1.18)	1.34 (1.15–1.55)
Colonoscopy	0.45 (0.41–0.49)	NA
EGD	NA	0.6 (0.55–0.64)
CCI	1.52 (1.46–1.58)	1.44 (1.39–1.5)
Ischemic coronary disease	0.75 (0.7–0.82)	0.73 (0.68–0.78)
Atrial fibrillation	0.78 (0.73–0.83)	0.85 (0.8–0.9)
Chronic liver disease	1.07 (0.97–1.18)	0.84 (0.75–0.95)
Angiodysplasia, *n* (%)	0.67 (0.58–0.77)	0.63 (0.54–0.73)
Acute renal failure	0.71 (0.65–0.76)	0.75 (0.69–0.8)
Type 2 diabetes	0.44 (0.41–0.48)	0.46 (0.43–0.5)
COPD	0.61 (0.55–0.66)	0.65 (0.6–0.71)
Colon cancer	1.57 (1.26–1.96)	1.67 (1.53–1.83)
Stomach cancer	1.91 (1.67–2.18)	1.78 (1.33–2.38)
Gastrointestinal bleeding	1.86 (1.74–1.99)	1.39 (1.31–1.49)
Inflammatory bowel disease	1.41 (0.86–2.32)	1.78 (1.47–2.16)
Red blood cell transfusion	1.1 (1.04–1.17)	1.04 (0.98–1.11)

EGD—esophagogastroduodenoscopy; CCI—Charlson Comorbidity Index; COPD—chronic obstructive pulmonary disease.

## Data Availability

Can be accessed from the Spanish Ministry of Health databases of the SNHDD, which provides it free of charge, in the following link: https://www.mscbs.gob.es/estadEstudios/estadisticas/estadisticas/estMinisterio/SolicitudCMBDdocs/2018_Formulario_Peticion_Datos_RAE_CMBD.pdf (accessed on 12 November 2020).

## Supplementary Materials

The following are available online at https://www.mdpi.com/2077-0383/10/3/546/s1. Table S1: Diagnosis and procedures analyzed with their corresponding ICD-9-CM and ICD-10 codes. Table S2: Sensitivity analysis. Distribution of study variables after propensity score matching according to the performance or non-performance of esophagogastroduodenoscopy after excluding patients with gastric and colorectal cancer. Table S3: Sensitivity analysis. Distribution of study variables after propensity score matching according to the performance or non-performance of colonoscopy after excluding patients with gastric and colorectal cancer.

## References

[1] Scott M.C., Winters M.E. (2015). Congestive Heart Failure. Emerg. Med. Clin. N. Am..

[2] Ezekowitz J.A., McAlister F.A. (2003). Armstrong PW-Anemia is common in heart failure and is associated with poor outcomes: Insights from a cohort of 12,065 patients with new-onset heart failure. Circulation.

[3] Groenveld H.F., Januzzi J.L., Damman K., van Wijngaarden J., Hillege H.L., van Veldhuisen D.J., van der Meer D. (2008). Anemia and Mortality in Heart Failure Patients- A Systematic Review and Meta-Analysis. J. Am. Coll. Cardiol..

[4] Sîrbu O., Floria M., Dascalita P., Stoica A., Adascalitei P., Sorodoc V., Sorodoc L. (2018). Anemia in heart failure-from guidelines to controversies and challenges. Anatol. J. Cardiol..

[5] Anand I.S., Gupta P. (2018). Anemia and Iron Deficiency in Heart Failure: Current Concepts and Emerging Therapies. Circulation.

[6] Yeo T.J., Yeo P.S., Wong R.C.-C., Ong H.Y., Leong K.T., Jaufeerally F., Sim D., Santhanakrishnan R., Lim S.L., MY Chan M. (2014). Iron deficiency in a multi-ethnic Asian population with and without heart failure: Prevalence, clinical correlates, functional significance and prognosis. Eur. J. Heart Fail..

[7] Martens P., Nijst P., Verbrugge F.H., Smeets K., Dupont M., Mullens W. (2017). Impact of iron deficiency on exercise capacity and outcome in heart failure with reduced, mid-range and preserved ejection fraction. Acta Cardiol..

[8] Cappellini M.D., Comin-Colet J., de Francisco A., Dignass A., Doehner W., Lam C.S., Macdougall I.C., Rogler G., Camaschella C., Kadir R. (2017). Iron deficiency across chronic inflammatory conditions: International expert opinion on definition, diagnosis, and management. Am. J. Hematol..

[9] Klip I.T., Comin-Colet J., Voors A.A., Ponikowski P., Enjuanes C., Banasiak W., Lok D.J., Rosentryt P., Torrens A., Polonski L. (2013). Iron deficiency in chronic heart failure: An international pooled analysis. Am. Heart J.

[10] Zain E.A.S., Mohammad A.G., Lobna A.W., Elham A.H., Khaled M.A. (2013). Upper Gastrointestinal Mucosal Changes in Patients with Congestive Heart Failure. Med. J. Cairo Univ..

[11] Romeiro F.G., Okoshi K., Zornoff L.A., Okoshi M.P. (2012). Gastrointestinal changes associated to heart failure. Arq. Bras. Cardiol..

[12] Ministry of Health Spanish National Hospital Discharge Database (Conjunto Minimo Basico de Datos). https://www.mscbs.gob.es/estadEstudios/estadisticas/cmbdhome.htm.

[13] Bonow R.O., Bennett S., Casey D.E., Ganiats T.G., Hlatky M.A., Konstam M.A., Lambrew C.T., Normand S.-L.T., Pina I.L., Radford M.J. (2005). ACC/AHA clinical performance measures for adults with chronic heart failure: A report of the American College of Cardiology/American Heart Association Task Force on Performance Measures (Writing Committee to Develop Heart Failure Clinical Performance Measures): Endorsed by the Heart Failure Society of America. Circulation.

[14] Mehta A.B., Syeda S.N., Wiener R.S., Walkey A.J. (2015). Epidemiological trends in invasive mechanical ventilation in the United States: A population-based study. J. Crit. Care.

[15] Quan H., Sundararajan V., Halfon P., Fong A., Burnand B., Luthi J.C., Saunders L.D., Beck C.A., Feasby T.E., Ghali W.A. (2005). Coding algorithms for defining comorbidities in ICD-9-CM and ICD-10 administrative data. Med. Care.

[16] Austin P.C. (2011). An Introduction to Propensity Score Methods for Reducing the Effects of Confounding in Observational Studies. Multivar. Behav. Res..

[17] D’Agostino R.B. (1998). Propensity score methods for bias reduction in the comparison of a treatment to a non-randomized control group. Stat. Med..

[18] Austin P.C. (2011). Comparing paired vs non-paired statistical methods of analyses when making inferences about absolute risk reductions in propensity-score matched samples. Stat. Med..

[19] Cosma A., Bănescub C., Mocan S., Balla B., Negovan A. (2019). Congestive Heart Failure and Upper Digestive Endoscopic Lesions. Acta Med. Marisiensis.

[20] Valkhoff V.E., Sturkenboom M.C., Kuipers E.J. (2012). Risk factors for gastrointestinal bleeding associated with low-dose aspirin. Best Pr. Res. Clin. Gastroenterol..

[21] García-Rayado G., Sostres C., Lanas A. (2017). Aspirin and Omeprazole for Secondary Prevention of Cardiovascular Disease in Patients at Risk for Aspirin-associated Gastric Ulcers. Expert. Rev. Clin. Pharmacol..

[22] Hasin T., Gerber Y., McNallan S.M., Weston S.A., Kushwaha S.S., Nelson T.J., Cerhan J.R., Roger V.L. (2013). Patients with heart failure have an increased risk of incident cancer. J. Am. Coll. Cardiol..

[23] Koene R.J., Prizment A.E., Blaes A., Konety S.H. (2016). Shared risk factors in cardiovascular disease and cancer. Circulation.

[24] Meijers W.C., Maglione M., Bakker S.J.L., Oberhuber R., Kieneker L.M., de Jong S., Haubner B.J., Nagengast W.B., Lyon A.R., van der Vegt B. (2018). Heart Failure Stimulates Tumor Growth by Circulating Factors. Circulation.

[25] Ponikowski P., Voors A.A., Anker S.D., Bueno H., Cleland J.G.F., Coats A.J.S., Falk V., González-Juanatey J.R., Harjola V.P., Jankowska E.A. (2016). 2016 ESC Guidelines for the diagnosis and treatment of acute and chronic heart failure: The Task Force for the diagnosis and treatment of acute and chronic heart failure of the European Society of Cardiology (ESC) Developed with the special contribution of the Heart Failure Association (HFA) of the ESC. Eur. Heart J..

[26] Yates J.M., Logan E.C., Stewart R.M. (2004). Iron deficiency anaemia in general practice: Clinical outcomes over three years and factors influencing diagnostic investigations. Postgrad. Med. J..

[27] Lucas C.A., Logan E.C., Logan R.F. (1996). Audit of the investigation and outcome of iron-deficiency anaemia in one health district. J. R Coll. Physicians Lond..

[28] Martens P., Minten L., Dupont M., Mullens W. (2019). Prevalence of underlying gastrointestinal malignancies in iron-deficient heart failure. ESC Heart Fail..

[29] Moratti R., Tramarin R., Tavazzi L. (2005). Blunted erythropoietin production and defective iron supply for erythropoiesis as major causes of anaemia in patients with chronic heart failure. Eur. Heart J..

[30] Van der Meer P., Voors A.A., Lipsic E., Smilde T.D., van Gilst W.H., van Veldhuisen D.J. (2004). Prognostic value of plasma erythropoietin on mortality in patients with chronic heart failure. J. Am. Coll. Cardiol..

[31] Grote Beverborg N., van Veldhuisen D.J., van der Meer P. (2018). Anemia in Heart Failure: Still Relevant?. JACC Heart Fail..

[32] Ponikowski P., Kirwan B.A., Anker S.D., McDonagh T., Dorobantu M., Drozdz J., Fabien V., Filippatos G., Göhring U.M., Keren A. (2020). Ferric carboxymaltose for iron deficiency at discharge after acute heart failure: A multicentre, double-blind, randomised, controlled trial. Lancet.

[33] Abu Ghanimeh M., Albadarinb S., Kaddourah O., Alturkmani H., Abughanimeh O., Tahboub M., Derbas L., Clarkston W. (2017). Safety of Gastrointestinal Endoscopic Procedures in Patients with Severe Heart Failure. Am. J. Gastroenterol..

[34] Sivananthan A., Glover B., Ayaru L., Patel K., Darzi A., Patel N. (2020). The evolution of lower gastrointestinal endoscopy: Where are we now?. Ther. Adv. Gastrointest Endosc..

[35] Graham D.G., Banks M.R. (2015). Advances in upper gastrointestinal endoscopy. F1000Research.

[36] ASGE Technology Committee (2014). High-definition and high-magnification endoscopes. Gastrointest. Endosc..

[37] Patel N., Seneci C., Yang G.-Z., Darzi A., Teare J. (2014). Flexible platforms for natural orifice transluminal and endoluminal surgery. Endosc. Int. Open.

[38] Spurr C., Sridhar S., Wu G. (2018). History of the instruments and techniques of gastrointestinal endoscopy. Diagnostic and Therapeutic Procedures in Gastroenterology: An Illustrated Guide.

[39] Manzano L., González-Franco Á., Cerqueiro J.M., Montero Pérez-Barquero M. (2017). Heart Failure Programs/Units. A Multidisciplinary Approach. Rev. Esp. Cardiol..

[40] Montminy E.M., Jang A., Conner M., Karlitz J.J. (2020). Screening for Colorectal Cancer. Med. Clin. N. Am..

