# Supplementary material for: Trends and Clinical Impact of Gastrointestinal Endoscopic Procedures on Acute Heart Failure in Spain (2002–2017)
Source: J Clin Med. 2021 Feb 2;10(3):546. doi: 10.3390/jcm10030546 (PMC7867243; doi:10.3390/jcm10030546)
Supplement: Supplementary file 1 [file jcm-10-00546-s001.pdf]

Trends and Clinical Impact of Gastrointestinal Endoscopic Procedures on Acute Heart Failure in Spain (2002-2017)

Manuel Méndez-Bailón, Rodrigo Jiménez-García, Nuria Muñoz-Rivas, Valentín Hernández-Barrera, José Maria de Miguel-Yanes, Javier de Miguel-Díez, Emmanuel Andrés, Noel Lorenzo-Villalba and Ana López-de-Andrés

Table S1. Diagnosis and procedures analyzed with their corresponding ICD-9-CM and ICD-10 codes.

Variable	ICD-9-CM	ICD-10
Heart failure	402.01, 402.11, 402.91, 404.01, 404.03, 404.11, 404.13, 404.91, 404.93, 428.12	I11.0, I13.0, I13.2, I125.5, I25.89, I25.9, I42, I42.x; I50; I50.xx
Esophagogastroduodenoscopy	44.13, 44.14, 44.43	0DJ68ZZ, 0D968ZX, 0W3P8ZZ, 0DQ68ZZ, 0DQ98ZZ
Colonoscopy	45.23, 45.24, 45.25, 48.23, 48.24, 48.36	0DJD8ZZ, 0DJD8ZZ, 0DBE8ZX, 0DBH8ZX, 0DBN8ZX, 0DDE8ZX, 0DDH8ZX, 0DDN8ZX, 0D9E8ZX, 0D9H8ZX, 0D9N8ZX, 0DBP8ZX, 0D9P8ZX, 0DBP8ZZ
Ischemic coronary disease	410.XX-414.XX	I20XX-I25XX
Atrial fibrillation	427.31	I48.0, I48.1, I48.2, I48.91
Anemia	285.2, 285.2x, 285.9	D63.0, D63.1, D63.8, D64.9
Chronic liver disease	570-573	K70-K77
Angiodysplasia	537.82, 537.83, 537.84, 569.84, 569.85, 569.86	K31.819, K31.811, K31.82, K55.20, K55.21, K63.81
Acute renal failure	584, 584.5, 584.6, 584.7, 584.8, 584.9	N17-N19
Type 2 diabetes	250.xx	E10-E14
COPD	491.21, 492.22, 491.8, 491.9, 492.8, 496	J40-J44
Colon cancer	153.x	C18.x
Gastric cancer	151.x	C16.x
Gastrointestinal bleeding	456.0, 456.20, 530.21, 530.7, 531.0, 531.2, 531.4, 531.6, 532.0, 532.2, 532.4, 532.6, 533.0, 533.2, 533.4, 533.6, 534.0, 534.2, 534.4, 534.6, 535.x1, 562.01, 562.02, 562.12, 562.13, 569.3, 578.X	I85.01, I85.11, K22.11, K22.6, K25.0, K25.2, K25.4, K25.6, K26.0, K26.2, K26.4, K26.6, K27.0, K27.2, K27.4, K27.6, K28.0, K28.2, K28.4, K28.6, K29.x1, K57.01, K57.11, K57.21, K57.31,K57.33, K57.41, K57.51, K57.53, K57.81, K57.91, K57.93, K62.5, K92.0, K92.1, K92.2
Inflammatory bowel disease	555.x, 556.x	K50.xx, K51.xx
Red cell transfusion	99.00, 99.01–99.08	30233H0, 30233N0, 30243H0,30243N0, 30253H0, 30253N0, 30263H0, 30263N0

COPD: Chronic obstructive pulmonary disease.

Table S2. Sensitivity analysis. Distribution of study variables after propensity score matching according to the performance or non-performance of esophagogastroduodenoscopy after excluding patents with gastric and colorectal cancer.

Variables	Esophagogastroduodenoscopy		No Esophagogastroduodenoscopy		*p*-value
	n	%	*n*	%	
2002–2003	4158	8.48	4382	8.94	0.019
2004–2005	4698	9.58	4912	10.02	
2006–2007	5699	11.62	5772	11.77	

2008–2009	6870	14.01	6869	14.01	
2010–2011	7263	14.81	7211	14.71	
2012–2013	7412	15.12	7206	14.70	
2014–2015	7331	14.95	7184	14.65	
2016–2017	5594	11.41	5489	11.20	
Female sex	24044	49.04	24081	49.12	0.813
Age, years, [mean] (SD)	[77.25]	[10.06]	[77.49]	[10.03]	<0.001
< 60 years	2658	5.42	2726	5.56	<0.001
60–75 years	13820	28.19	13216	26.96	
76–85 years	23612	48.16	23614	48.17	
> 85 years	8935	18.23	9469	19.31	
Colonoscopy	15199	31.00	15451	31.52	0.082
CCI, [mean] (SD)	[2.43]	[1.05]	[2.36]	[1.04]	<0.001
Ischemic coronary disease	11154	22.75	10305	21.02	<0.001
Atrial fibrillation	20949	42.73	21214	43.27	0.087
Anemia	289	0.59	302	0.61	0.592
Chronic liver disease	4414	9.00	4580	9.34	0.066
Angiodysplasia	3661	7.47	2974	6.07	<0.001
Acute renal failure	12716	25.94	12306	25.10	0.002
Type 2 diabetes	16802	34.27	15769	32.17	<0.001
COPD	8783	17.92	7996	16.31	<0.001
Gastrointestinal bleeding	10168	20.74	10532	21.48	0.004
Inflammatory bowel disease	159	0.32	170	0.35	0.543
Red cell transfusion	19057	38.87	19899	40.59	<0.001
LOHS, [mean] (SD)	[17.25]	[16.35]	[14.41]	[13.95]	<0.001
IHM, *n* (%)	4272	8.71	6819	13.91	<0.001

CCI: Charlson Comorbidity Index, COPD: Chronic obstructive pulmonary disease, LOHS: Length of hospital stay, IHM: In hospital mortality.

Table S3. Sensitivity analysis. Distribution of study variables after propensity score matching according to the performance or non-performance of colonoscopy after excluding patents with gastric and colorectal cancer.

Variables	Colonoscopy		Colonoscopy		*p*-value
	n	%	*n*	%	
2002–2003	4455	6.65	4870	7.27	<0.001
2004–2005	5722	8.54	6054	9.04	
2006–2007	7555	11.28	7556	11.28	
2008–2009	8925	13.32	8752	13.06	
2010–2011	10285	15.35	9771	14.58	
2012–2013	10399	15.52	10086	15.05	
2014–2015	10651	15.90	10371	15.48	
2016–2017	9006	13.44	9539	14.24	
Female sex	33822	50.48	33438	49.91	0.036
Age, years, [mean] (SD)	[76.95]	[10.16]	[77.40]	[10.11]	<0.001
< 60 years	2961	4.42	3408	5.09	<0.001
60–75 years	19225	28.69	18441	27.52	
76–85 years	33283	49.68	32285	48.19	
> 85 years	11530	17.21	12865	19.20	
Esophagogastroduodenoscopy	15166	22.64	13736	20.50	<0.001
CCI, [mean] (SD)	[2.25]	[1.07]	[2.19]	[1.06]	<0.001

Ischemic coronary disease	15758	23.52	14570	21.75	<0.001
Atrial fibrillation	28996	43.28	28490	42.52	0.005
Anemia	480	0.71	494	0.74	0.652
Chronic liver disease	4032	6.02	4305	6.43	0.002
Angiodysplasia	3879	5.79	3540	5.28	<0.001
Acute renal failure	17786	26.55	17619	26.30	0.301
Type 2 diabetes	23796	35.52	22895	34.17	<0.001
COPD	12326	18.40	11756	17.55	<0.001
Gastrointestinal bleeding	13347	19.92	13636	20.35	0.049
Inflammatory bowel disease	922	1.38	1102	1.65	<0.001
Red cell transfusion	23758	35.46	24775	36.98	<0.001
LOHS, [mean] (SD)	[16.845]	[14.02]	[14.03]	[1421]	<0.001
IHM, *n* (%)	5093	7.60	10356	15.46	<0.001

CCI: Charlson Comorbidity Index, COPD: Chronic obstructive pulmonary disease, LOHS: Length of hospital stay, IHM: In hospital mortality.
